# 

**Published:** 1895-04-27

**Authors:** 


					- "inn mnu ur iwiukhl iHDit WAitKS. mt 1 ? yy
" "inn Miiu ur nhiuiim. iholc w
tloftaiittu tH|
M
uuZTnBrwich hospital
No. 448. Vol. XTm with EXTRA NURSING SUPPLEMENT.
^ATURD \V a prtt 97ttt 1 RQ z, [Registered at the General Post Offioe as a Nowsyaper for Montlily Parts, 6d.; post free, 8<i.
' ? trammissiou in the United Kingdom and Abroad.] Dittoper Annum, 8f. 6d.,post free.

				

